# Testing the Purity of the Atmosphere

**Published:** 1883-10

**Authors:** 


### Testing the Purity of the Atmosphere.

A plain and simple test, by which any layman would be able to say if the air of a room can still be inhaled without any detriment to the human organism, has still so far been a desideratum. Long ago it has been proven that for the purpose of deciding such a question it is necessary only to determine the percentage of carbonic acid the air contains. For practical purposes it is utterly superfluous to find out what chemical agents or what animaliculæ or molecular changes may have altered the air to be respired; because Pettenkofer has proven beyond a doubt that any air containing more than one pro mill of carbonic acid—speaking of the common atmosphere of the room—is hurtful to breathe continually, and that a perfectly innocuous air, in which a human being can live comfortably for a long time without harm, should not possess more carbonic acid than seven-tenths pro mill.

Dr. Wolpert (*Monatsch. f. Med. Polytechni*, July 1, 1883,) has invented a simple apparatus, for this purpose, and the same is placed the first time for public inspection in the Berlin Hygienic Exposition of this year. The apparatus consists of a gum ball, a small glass tube fitting into the same, and a common reaction glass, which has a mark ground into it to show how far it should be filled, and the bottom of which consists of milk glass (or glass otherwise clouded) with a bright test-mark, which in this case is the year 1883, *i. e.*, the whole glass and these numbers are clear glass, only the glass surrounding at the bottom of the figures is clouded. The test depends upon the well-known effect of carbonic acid upon lime-water. If the test be made in a certain room, the reaction-glass is filled up to the mark with lime-water; the compressed gum ball is permitted to fill itself with the air of the room through the glass tube inserted, then the latter is placed into the fluid in the test tube, and the air of the ball expelled into the fluid. While in any good air it is necessary to expel the air from the always refilled gum ball into the fluid of test-tube twenty to thirty times, two to three times are sufficient if the air is bad to make the lime-water so cloudy that the test sign cannot be read any more. A tabular statement accompanies the whole, from which, according to the number of times the air of the gum ball had to be expelled into the fluid ere the latter became sufficiently cloudy to obscure the test sign, the percentage of carbonic acid the air contains can be determined. For practical purposes, it is sufficient to remember that if the lime-water becomes turbid after ten fillings the air is too impure to be breathed without detriment. If from ten to twenty fillings are needed, the air may be inhaled for a short time; if more than twenty, it is a sign that the air may be considered good.

Certainly, everybody may provide himself with a similar test if he possesses a number of vessels containing a certain known percentage of carbonic acid, and noting the effect of such air each time upon a test tube filled with lime-water. If a common test tube is filled four-fifths with pure lime-water, and if then a common gum ball is taken, compressed, and a person while gradually yielding the pressure, fills the ball with the air he expires, and if then this air is expelled into the fluid of the test tube, and if this fluid then, after being shaken, becomes so turbid that a bright sign on the clouded bottom cannot be read, in that case it can be taken for granted that the air contained four per cent of carbonic acid (as well known). From this effect on the lime-water, a smaller percentage may be calculated, but a better and surer procedure for one who would wish to manufacture such a handy and valuable apparatus for himself, would always be to make his first observations with air containing known definite quantities of carbonic acid, and then to make his calculation therefrom.—*Medical and Surg. Reporter.*

